# Antiviral Activity of Ribavirin against *Tilapia tilapinevirus* in Fish Cells

**DOI:** 10.3390/pathogens10121616

**Published:** 2021-12-10

**Authors:** Tuchakorn Lertwanakarn, Pirada Trongwongsa, Sangchai Yingsakmongkol, Matepiya Khemthong, Puntanat Tattiyapong, Win Surachetpong

**Affiliations:** 1 Department of Physiology, Faculty of Veterinary Medicine, Kasetsart University, Bangkok 10900, Thailand; fvettol@ku.ac.th; 2 Department of Veterinary Microbiology and Immunology, Faculty of Veterinary Medicine, Kasetsart University, Bangkok 10900, Thailand; piradatr@gmail.com (P.T.); fvetscy@ku.ac.th (S.Y.); matepiya@hotmail.com (M.K.); puntanat.t@ku.th (P.T.); 3 Interdisciplinary Genetic Engineering Program, The Graduate School, Kasetsart University, Bangkok 10900, Thailand

**Keywords:** *Tilapia tilapinevirus*, Tilapia lake virus, antivirus, ribavirin, tilapia, TiLV

## Abstract

The outbreak of the novel *Tilapia tilapinevirus* or Tilapia lake virus (TiLV) is having a severe economic impact on global tilapia aquaculture. Effective treatments and vaccines for TiLV are lacking. In this study, we demonstrated the antiviral activity of ribavirin against TiLV in E-11 cells. Our findings revealed that at concentrations above 100 μg/mL, ribavirin efficiently attenuates the cytopathic effect of the TiLV infection in fish cells. When administered in a dose-dependent manner, ribavirin significantly improved cell survival compared to the untreated control cells. Further investigation revealed that the cells exposed to ribavirin and TiLV had a lower viral load (*p* < 0.05) than the untreated cells. However, at concentrations above 1000 μg/mL, ribavirin led to cell toxicity. Taken together, our results demonstrate the efficacy of this antiviral drug against TiLV and could be a useful tool for future research on the pathogenesis and replication mechanism of TiLV as well as other piscine viruses.

## 1. Introduction

Tilapia lake virus (TiLV), or *Tilapia tilapinevirus*, is the etiological agent of Tilapia lake virus disease (TiLVD), which is having a severe economic impact on the global tilapia industry [1]. To date, infection with TiLV has been reported in 16 countries [1,2]. TiLV is a single-stranded, negative-sense RNA virus, categorized in the new family, *Amnoonviridae*. Its genome size is approximately 10,323 bp, containing 10 segments, which encode for 14 proteins [1]. Only segment 1 of the virus shares a weak sequence homology with the end of the polymerase of the influenza C virus [3]. TiLV can cause disease in various fish species, including hybrid tilapia (*Oreochromis niloticus* × *O. aureus*), Nile tilapia (*O. niloticus*), grey tilapia (*O. niloticus* × *O. aureus*), red tilapia (*Oreochromis* sp.), Mozambique tilapia (*O. mossambicus*) [4,5,6,7,8,9,10], giant gourami (*Osphronemus goramy*) [11,12], and ornamental African cichlids (*Aulonocara* spp.) [13]. The clinical signs and gross pathology of infected fish are anorexia, abnormal swimming, severe anemia, exophthalmia, skin erosion and congestion, scale protrusion, and abdominal swelling. Interestingly, fish that survive TiLVD develop a protective immunity partly through antibody production that prevents them from later infection [14].

Currently, the economic impact and damage caused by TiLV can be lessened through strict biosecurity practices [1]. For example, most common aquaculture disinfectants can inactivate TiLV at 28 °C in less than 1–5 min exposures [15]. Additionally, probiotic supplementation can alleviate disease severity and improve the survival of fish during TiLV infection [16]. Nevertheless, the lack of effective treatments and vaccines for TiLV points to the importance of further research and a knowledge gap that needs to be filled.

Ribavirin is a synthetic nucleoside analog that possesses antiviral effects against different DNA and RNA viruses such as adenovirus, paramyxovirus, hantavirus, coronavirus, Lassa virus, and influenza virus [17,18,19,20]. The proposed mechanisms of ribavirin include the inhibition of inosine monophosphate dehydrogenase (IMPDH), which interferes with the production of purine nucleosides and further suppresses DNA and RNA synthesis [17,21,22], mimics 7-methyltguanosine cap leading to the inhibition of viral mRNA translational and capping process of the viruses [23,24], and blocks viral RNA polymerase [25,26]. Previously, ribavirin has been studied extensively in a large number of mammals and aquatic species [27,28,29]. In fish, ribavirin prevents infectious salmon anemia virus (ISAV) replication through the inhibition of RNA synthesis [30]. Additionally, ribavirin targets guanidine nucleotides synthesis and inhibits viral hemorrhagic septicemia virus transcription in salmonid cells [31]. Although ribavirin has been shown to be effective in a variety of aquatic viruses, the efficacy of ribavirin against tilapia viruses has not yet been investigated. In this study, we investigated the antiviral effects of ribavirin against TiLV in fish cells.

## 2. Results

### 2.1. Toxicity Effect of Ribavirin on E-11 Cells

**Figure 1:**
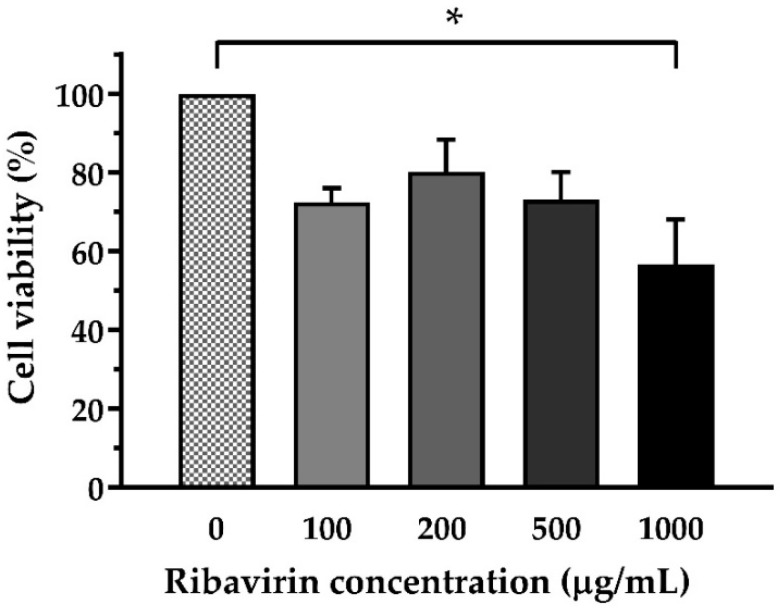
Toxicity of ribavirin against E-11 cells. The E-11 cells were treated with ribavirin at concentrations in the range 100–1000 µg/mL and then evaluated for cell survival at 7 days using a Cell Counting Kit-8 assay. The data represent the mean cell viability ± SEM from 3 replicates and the comparisons carried out using one-way ANOVA and Dunnett’s multiple comparison test. Significance (*p* < 0.05) is marked as *.

First, the cytotoxicity effect of ribavirin on E-11 cells was evaluated using a CCK-8 assay. In this experiment, the cells were incubated with ribavirin at a concentration of 100−1000 μg/mL for 7 days. The survival rates of the E-11 cells exposed to ribavirin at 100, 200, and 500 μg/mL were 72.42% ± 3.59%, 80.15% ± 8.20%, and 73.11% ± 7.00%, respectively (Figure 1). Remarkedly, ribavirin at 1000 μg/mL caused extensive cell death with the percentage of cell survival being 56.65% ± 11.46% compared to the control group (*p* < 0.05).

### 2.2. Ribavirin Reduced TiLV-Induced Cytopathic Effect in E-11 Cells

**Figure 2:**
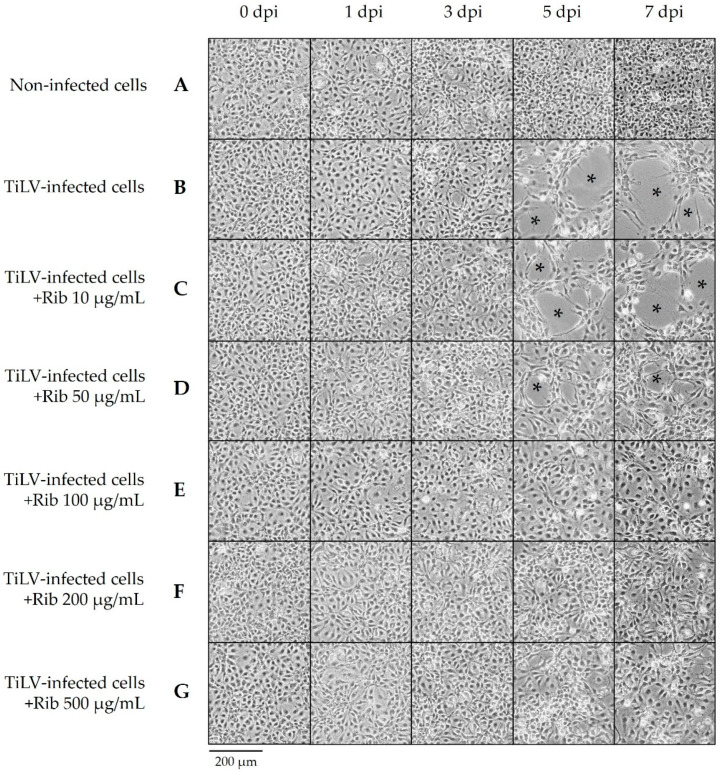
Ribavirin inhibited TiLV-induced CPE formation in E-11 cells in a dose-dependent concentration. Confluent E-11 cells were incubated with TiLV at a multiplicity of infection (MOI) of 0.46 for 1 h followed by ribavirin treatment. (**A**) Non-infected cells; mock-infected E-11 cells treated with an L-15 medium. (**B**) TiLV-infected cells without ribavirin. (**C**–**G**) The TiLV-infected E-11 cells were treated with serial dilutions of ribavirin at 10, 50, 100, 200, and 500 µg/mL. The cytopathic effect (CPE) formation (*) was monitored daily until 7 days post-infection (dpi).

The morphological appearance of the E-11 cells incubated with TiLV and/or ribavirin is demonstrated in Figure 2. Compared to the non-infected control cells, the E-11 cells incubated with TiLV showed a distinct cytopathic effect (CPE) between 5 and 7 days post-infection (dpi) (Figure 2B). Notably, ribavirin attenuated CPE formation induced by the TiLV infection, in a dose-dependent concentration (Figure 2C–G). Infected E-11 cells treated with ribavirin above 100 μg/mL showed a normal cell morphology until 7 dpi (Figure 2D–G). In contrast, the infected cells treated with ribavirin at 10 and 50 μg/mL developed CPE formation at 5 dpi (Figure 2A–D). However, less CPE formation was observed in the infected cells treated with 50 μg/mL ribavirin.

### 2.3. Ribavirin Treatment Improves Cell Viability during TiLV Infection

**Figure 3:**
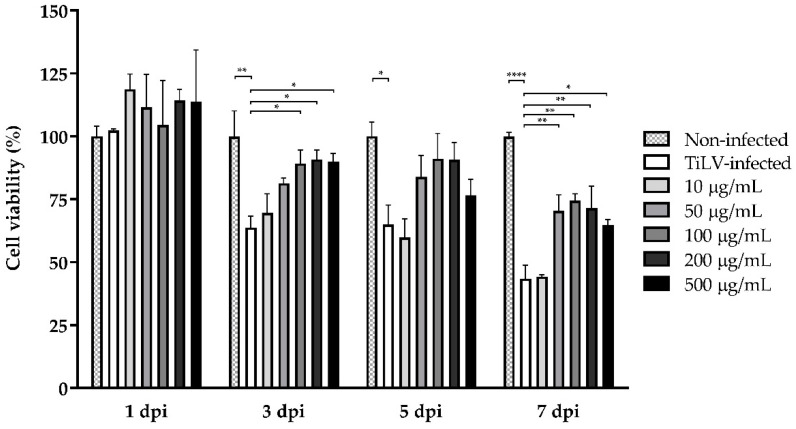
Survival of infected E-11 cells incubated with different ribavirin concentrations at 1, 3, 5, and 7 dpi. The percentage of surviving cells between the ribavirin-treated and sham-treated group was compared using two-way ANOVA followed by Bonferroni’s multiple comparison test. In the sham-treated group, the cell viability was reduced chronologically. Treating with ribavirin ranging from 100 to 500 μg/mL significantly improved the cell survival in a dose-dependent manner. The data are represented as mean + SEM from three replicates. Significance is indicated as * for *p* < 0.05, ** for *p* < 0.01, and **** for *p* < 0.0001.

To further quantify the viability of E-11 cells after TiLV infection, the infected cells with ribavirin and sham (diluent) treatments were examined using the CCK-8 assay. The uninfected cells showed 100% survival during the entire experiment (Figure 3), while the TiLV-infected E-11 cells had survival rates of 63.77% ± 4.51%, 64.96% ± 7.77%, and 43.47% ± 5.26% at 3, 5, and 7 dpi, respectively. At low ribavirin concentrations (10 μg/mL), the survival of the infected E-11 cells was 69.57% ± 7.53%, 59.85% ± 7.38%, and 44.27% ± 0.84% at 3, 5, and 7 dpi, respectively, while treatment with 50 μg/mL resulted in 81.37% ± 1.99%, 83.90% ± 8.54%, and 70.38% ± 6.44% survival rates at 3, 5, and 7 dpi, respectively. Interestingly, ribavirin at 100 μg/mL statistically improved cell viability at 3 and 7 dpi, with the survival rates being 89.23% ± 5.32% (*p* < 0.05) and 74.52% ± 2.63% (*p* < 0.01). Likewise, the infected cells treated with ribavirin at 200–500 μg/mL had less cell death and better survival rates of between 90.06 and 90.89% ± 3.19–3.73% (*p* < 0.05) at 3 dpi and 64.81 and 71.50% ± 2.07–8.73% (*p* < 0.05) at 7 dpi than the sham-treated cells.

### 2.4. Lower TiLV RNA in Ribavirin-Treated E-11 Cells

**Figure 4:**
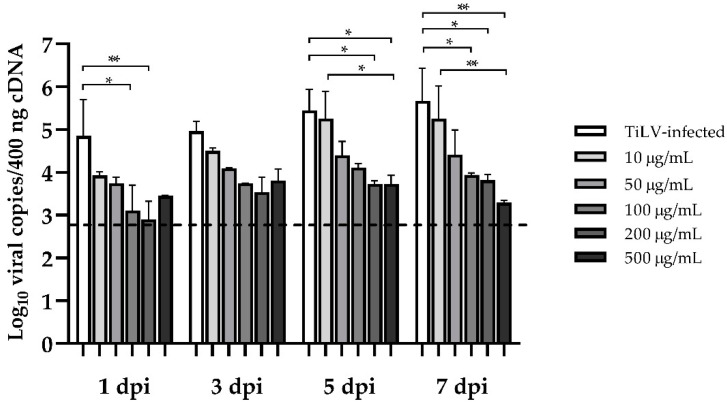
Effect of ribavirin treatment on TiLV RNA concentrations in E-11 cells. The cells were incubated with TiLV for 1 h followed by ribavirin at concentrations ranging from 10 to 500 μg/mL. The mean log_10_ viral copies between the ribavirin-treated cells and the sham (diluent)-treated cells were compared using two-way ANOVA followed by Bonferroni’s multiple comparison test. The data are represented as mean ± SEM from 3 replicates. Significance is indicated as * for *p* < 0.05 and ** for *p* < 0.01. The cut-off limit (dot line) was set at Ct 34, which equals 10^2.84^ viral copies per 400 ng cDNA.

The amount of TiLV RNA in the ribavirin-treated and TiLV-infected control (sham) cells was further investigated. The infected E-11 cells without ribavirin treatment had 10^4.85 ± 0.85^ viral copies per 400 ng cDNA within 24 h of virus exposure, and this gradually increased to 10^5.67 ± 0.76^ after 7 dpi (Figure 4). Treatment with low concentrations of ribavirin (10 and 50 μg/mL) showed no significant change in TiLV RNA in the E-11 cells at all time points, with a range of 10^3.75 ± 0.14^ to 10^5.25 ± 0.77^ viral copies per 400 ng cDNA. Notably, the infected cells treated with high ribavirin concentrations (100–500 μg/mL) showed a dose-dependent reduction in TiLV RNA concentrations at 1 dpi (10^2.90 ± 0.42^ to 10^3.45 ± 0.10^), 5 dpi (10^3.73 ± 0.2^ to 10^4.11 ± 0.10^), and 7 dpi (10^3.29 ± 0.05^ to 10^3.94 ± 0.05^), respectively.

## 3. Discussion

Tilapia is considered one of the most important protein sources in the 21st century [32]. Indeed, tilapia is the second most important freshwater fish cultured worldwide, with global production at 6.4 million tons in 2015 [33]. Over the past few years, outbreaks of emerging viruses such as TiLV have put the global tilapia farming industry at risk and have challenged sustainable production in the industry [1,34,35]. Despite the considerable interest in, and multiple attempts to develop, an effective vaccine for TiLV [36,37,38,39], no commercial vaccines are available to prevent fish from contracting TiLV. Hence, alternative studies on therapeutics or compounds to control TiLV outbreaks, especially antiviral agents, are urgently needed. This study presents the first evidence of the application of the antiviral agent ribavirin against TiLV in fish cells in vitro. We demonstrated that ribavirin treatment reduces CPE formation and TiLV RNA concentrations and improves cell survival.

Previous studies have shown that TiLV infection can lead to rapid CPE formation and massive cell death within 3–7 days in various fish cell lines [5,40,41,42,43,44]. In this study, TiLV causes dramatic morphological changes and complete cell destruction within 5 days. However, ribavirin attenuated cell death in a dose-dependent manner and improved the viability of E-11 cells after TiLV exposure. Notably, there were no statistically significant differences between the sham-treated and infected cells treated with various ribavirin concentrations at 5 dpi. It could be explained that the variation between the replicates could partly affect the statistical analysis, showing nothing significant at this time point. However, a pattern of viral reduction was also observed when cells were exposed to ribavirin at the concentration above 100 µg/mL, which is consistent with the results found at 3 dpi and 7 dpi. Apart from the viral infection, several factors may contribute to the lower survival rate of E-11 cells at 7 dpi, such as the loss of action of the compound. Nonetheless, a combination of observing cell morphology under a microscope and assessing the cell viability using the CCK-8 assay revealed that ribavirin at the concentration above 100 µg/mL could prevent cell death from TiLV infection. In other studies, ribavirin inhibited ISAV replication and significantly reduced ISAV viral loads, and CPE formation in salmon head kidney (SHK-1) cell lines [30]. Similarly, ribavirin reduced CPE formation and viral loads in cells incubated with infectious hematopoietic necrosis virus, infectious pancreatic necrosis virus, and viral hemorrhagic septicemia virus [31,45,46]. Although we demonstrated that ribavirin could improve cell survival against TiLV infection, a high ribavirin concentration of 1000 μg/mL had a strong negative impact on the survival of the E-11 cells. Likewise, high concentrations of ribavirin have toxic effects on other fish cell lines, including Chinook salmon embryonic (CHSE-214) and rainbow trout gonad (RTG-2) cells [47], flounder spleen cells [46], salmon head kidney cells (SHK-1) [48], and carp Epithelioma papulosum cyprini cells (EPC) [31]. Based on our results indicating its toxicity effect on E-11 cells and the efficacy of ribavirin against TiLV, we recommend ribavirin at 100–500 μg/mL to study the interaction of TiLV and fish cells.

Ribavirin is a nucleoside analogue, which is well known for its broad-spectrum antiviral activity [21]. This antiviral agent has been applied to study and prevent the infection of DNA and RNA viruses, such as adenovirus, paramyxovirus, hantavirus, coronavirus, Lassa virus, and influenza virus, in terrestrial animals and humans [17,18,19,20]. The mechanisms of ribavirin to inhibit RNA viral replication include the inhibition of IMPDH (yellow fever and hepatitis C viruses) [17], the blockade of the mRNA translational process via ribavirin triphosphate and its active metabolite (Lassa fever and the SARS coronaviruses) [24], and the interference of RNA polymerase enzymes (ISAV, influenza A, and HIV-1) [26,30]. Since TiLV has an RNA genome that is similar to those of these RNA viruses, we speculated that the antiviral effect of ribavirin on TiLV in E-11 cells may act via similar mechanisms as the aforementioned. Nevertheless, the mechanisms of ribavirin against TiLV replication need further investigation. Our study is the first to report the antiviral effect of ribavirin against TiLV and introduces the possibility of applying this drug or relevant compounds as tools to understand the pathogenesis and host–virus interaction in tilapia. In other fish models, ribavirin treatment reduced mortality in zebrafish larvae after nervous necrosis virus infection [28]. Moreover, ribavirin has an antiviral effect against Micropterus salmoides rhabdovirus by blocking viral particle release, reducing cell death, and improving cell survival [49]. In Atlantic salmon, ribavirin stimulates the expression of genes participating in T-helper pathways, such as IFN-γ and CD4 [48,50]. Notwithstanding, the application of this antiviral agent in tilapia in vivo needs further investigation. Certainly, ribavirin could serve as a good positive control to elucidate the pathogenesis and interaction of TiLV with fish cells.

## 4. Materials and Methods

### 4.1. Cell Culture and Virus

E-11 cells, a clone of SSN-1 cells [51] originating from snakehead fish (Ophiocephalus striatus), were purchased from the European Collection Authenticated Cell Cultures (ECACC), England (catalogue number 01110916), and were maintained at 25 °C without CO_2_ in Leibovitz’s L-15 medium supplemented with 2 mM L-glutamine pH 7.4 (L-15) (L4386, Sigma–Aldrich, St. Louis, MO, USA) and fetal bovine serum (FBS) (Thermo Fisher Scientific Inc., Waltham, MA, USA) at either 5% (vol/vol) for routine cell culture or 2% (vol/vol) for virus propagation. The confluent cells were routinely split by removing the cell culture media and then washed twice with sterile phosphate-buffered saline (PBS). The cells were split at a ratio 1:2 using 0.125% trypsin/EDTA. The resuspended cells were transferred to new cell culture flasks or plates at a density of 3 × 10^4^ cells/cm2 and incubated at 25 °C.

The TiLV strain VETKU-TV01 was isolated from moribund red hybrid tilapia (Oreochromis spp.) obtained from a commercial fish farm in Pathum Thani province, Thailand [50]. The virus was routinely propagated in E11 cells in L-15 with 2% FBS at 25 °C with an MOI of 0.1–0.5. At 80% cytopathic effect (CPE) formation, the infected cells were collected by freeze/thawing and centrifuged at 3000× *g* for 10 min. The supernatant was aliquoted in a 1.5 mL tube and stored at −80 °C for subsequent use. The viral titer was determined by a tissue culture infectious dose (TCID_50_/mL) assay according to the method described by Reed and Muench [52,53].

### 4.2. Ribavirin Dilution

A vial containing 10 mg ribavirin (catalog number R9644, Sigma-Aldrich, St. Louis, MO, USA) was resuspended with 1 mL autoclaved ultrapure (18.2 MΩ-cm resistivities) water from ELGA PURELAB Ultra Genetic (VWS Deutschland, Berlin, Germany). The final concentration of ribavirin was freshly prepared with the serial dilution of stock solutions (10,000 µg/mL) to 500, 200, 100, 50, and 10 µg/mL using an L-15 medium supplemented with 5% FBS.

### 4.3. Ribavirin Cytotoxicity Assay

The E-11 cells were plated at 9000 cells/well in 100 µL L-15 with 5% FBS in a 96-well microplate. After reaching 80–90% confluency, the cells were treated with ribavirin at concentrations of 0, 100, 200, 500, and 1000 µg/mL (three replicates/concentration) and kept at 25 °C for 7 days. The cell morphology was examined daily under a microscope, and the cell viability was assessed using the Cell Counting Kit-8 (CCK-8) assay described in Section 4.4. The mean 450 nm absorbance (A_450_) of the ribavirin-treated samples was compared to the mean A_450_ of the control samples without ribavirin. The cytotoxicity assay was repeated 3 times in different experimental setups.

### 4.4. CCK-8 Assay

The cell viability was measured using the CCK-8 assay (catalog number 96992, Sigma-Aldrich, St. Louis, MO, USA) according to the manufacturer’s instructions, albeit with a slight modification. Briefly, 10 µL of the CCK-8 solution was added directly to each sample containing 100 µL L-15 media in a 96-well microplate and incubated at 25 °C for 4 h. The reaction was then measured using a microplate reader (BioTek™ Synergy™ H1, Fisher Scientific, Leicestershire, UK) at A_450_. The cell viability was compared between the treatment groups containing ribavirin or TiLV, and the control groups without ribavirin or the virus.

### 4.5. Ribavirin Antiviral Effect

The confluent E-11 cells in 24-well plates were washed twice with L-15 media without FBS. For the TiLV infection, 300 µL of viral stock containing the TiLV strain VETKU-TV01 with 1.59 × 10^5^ TCID_50_/mL (equivalent to MOI 0.46) was incubated at 25 °C for 1 h. An equal volume of L-15 media without FBS or TiLV was added to the negative control wells. After 1 h, the media was substituted with 500 µL L-15 containing 2% FBS and ribavirin at 0, 10, 50, 100, 200, and 500 µg/mL. For the control groups, the cells were incubated with 2% FBS L-15 without ribavirin. The CPE formation was observed daily under a microscope. On day 0 (after 1 h incubation), 1, 3, 5, and 7 post-infection samples were collected in a 24-well plate by freeze/thawing. The samples were stored at −80 °C for subsequent TiLV quantification using reverse transcription quantitative polymerase chain reaction (RT-qPCR). In a separate experiment, a similar setup was performed in 96-well plates with 3 replicates/groups for the cytotoxicity assay, as described in Section 4.3.

### 4.6. RNA Extraction, Cdna Synthesis, and RT-Qpcr

Three hundred microliters of the samples described in Section 4.5 were added to 900 µL of ice-cold GENEzol^TM^ Reagent (Geneaid, Taiwan). The samples were then mixed with 180 µL of chloroform (Sigma-Aldrich, St. Louis, MO, USA) and incubated at room temperature for 3 min. The samples were centrifuged at 12,000× *g* at 4 °C for 15 min (Centrifuge 5418 R; Eppendorf, Hamburg, Germany). The supernatant was transferred to a new tube and mixed with 1 µL DNase I (Thermo Fisher Scientific, Waltham, MA, USA) and incubated at 37 °C for 30 min. An equal amount of 2-propanol (Merck, Darmstadt, Germany) was added to the samples, which were stored at −20 °C for 2 h. The samples were then centrifuged at 12,000× *g* at 4 °C for 15 min to collect the RNA pellet. After discarding the supernatant, the RNA pellet was washed in 1 mL 75% ethanol, then air-dried after being centrifuged at 12,000× *g* at 4 °C for 15 min. The RNA pellet was dissolved in 40 µL diethylpyrocarbonate (DEPC)-treated water. The amount of RNA was measured using NanoDrop (NanoDrop2000; Thermo Fisher Scientific Inc., Waltham, MA, USA).

For the cDNA synthesis, 200 ng/µL of RNA was converted to cDNA using ReverTraAce™ qPCR RT MasterMix (Toyobo, Osaka, Japan) according to the manufacturer’s instructions. The 20 µL reaction contained 4 µL DEPC-treated water, 4 µL 5 × RT buffer, 1 µL primer mix, 1 µL enzyme mix, and 10 µL RNA sample (2000 ng). The reaction was carried out in a thermocycler (T100 PCR thermocycler; Bio-Rad Laboratories, Hercules, CA, USA) at 42 °C for 60 min, 98 °C for 5 min, and held at 4 °C until collection.

A SYBR green-based RT-qPCR assay targeting segment 3 of the virus was used to determine the TiLV viral copy [54]. Briefly, 4 µL of cDNA (100 ng/µL) was added to a 6 µL master mix containing 5 µL 2 × iTaq™ Universal SYBR^®^ Green Supermix (Bio-Rad Laboratories, Hercules, CA, USA), 0.3 µL of 10 µM TiLV-112F forward primer (5′-CTGAGCTAAAGAGGCAATATGGATT-3′), 0.3 µL of 10 µM TiLV-112R reverse primer (5′-CGTGCGTACTCGTTCAGTATAAGTTCT-3′), and 0.4 µL of molecular-grade water. The qPCR reactions were performed in duplicate in a CFX96^®^ Touch thermal cycler (Bio-Rad Laboratories, Hercules, CA, USA). The cycling conditions at 98 °C for 3 min were the initial activation step and were followed by 40 cycles of 95 °C for 10 s and 60 °C for 30 s. At the end of the cycle, the reaction was heated from 65 °C to 95 °C at a rate of 0.5 °C per 5 s to confirm the melting temperature of the product. A no-template control was included in the test. The log copy number of the TiLV virus in the samples was extrapolated from the standard curve established from 10-fold serial dilutions of the pTG19-T plasmid (Vivantis, Shah Alam, Malaysia) containing a 491 bp TiLV segment 3 [54].

### 4.7. Statistical Analysis

The data were analyzed using GraphPad Prism software version 5.01 (GraphPad Software, San Diego, CA, USA). The mean percentage of the cell viability between the treatment and control groups was compared using one-way ANOVA followed by post hoc Dunnett’s multiple comparison test. The mean log_10_ viral copies in the antiviral assay were analyzed by two-way ANOVA followed by Bonferroni’s multiple comparison tests to compare the means among the sample groups. A *p*-value less than 0.05 was considered statistically significant.

## 5. Conclusions

Our study provides the first evidence that ribavirin efficiently attenuates the cytopathic effect caused by TiLV infection in fish cells. Moreover, we found that ribavirin inhibits TiLV replication and improves cell survival in a dose-dependent manner. Notwithstanding, we noted that at high concentrations, ribavirin can lead to cell toxicity in E-11 cells. Overall, our study demonstrates the efficacy of antiviral drugs against TiLV. This new knowledge could be applied as a tool in future studies on the pathogenesis and replication mechanism of this emerging virus. Further in vivo research is important to demonstrate the efficacy of ribavirin in tilapia during TiLV infection. The findings obtained in this study may contribute to the further development of antiviral agents against TiLV and other fish viruses.

## Data Availability

The data that support the findings of this study are available from the corresponding author upon reasonable request.
